# Glial Activation Enhances Spinal TRPV1 Receptor Sensitivity in a Paclitaxel Model of Neuropathic Pain

**DOI:** 10.33549/physiolres.935599

**Published:** 2025-08-01

**Authors:** Jakub SLEPICKA, Jiri PALECEK

**Affiliations:** 1 Laboratory of Pain Research, Institute of Physiology of the Czech Academy of Sciences, Prague, Czech Republic; 2 Charles University, Faculty of Science, Department of Physiology, Prague, Czech Republic

**Keywords:** Pain, Paclitaxel, Neuropathy, Glia, Minocycline, Synaptic transmission

## Abstract

Paclitaxel (PTX), a commonly used chemotherapeutic, frequently leads to chemotherapy-induced peripheral neuropathy (CIPN), characterized by persistent pain and neuronal hypersensitivity. While its effects on peripheral nerves are well-documented, paclitaxel also influences central nervous system pathways, particularly spinal synaptic transmission, through Toll-like receptor 4 (TLR4) activation and subsequent sensitization of transient receptor potential vanilloid 1 (TRPV1) receptors. In this study, we used an in vitro model of paclitaxel-induced neuropathic pain to investigate the role of glial activation in TRPV1 receptor function.

Using whole-cell patch-clamp recordings from superficial dorsal horn neurons in acute spinal cord slices, we evaluated the effects of minocycline (MX), a glial cell inhibitor, and ISO-1, a macrophage migration inhibitory factor (MIF) antagonist, on paclitaxel-induced synaptic changes. Our results demonstrate that acute paclitaxel application enhances nociceptive signaling and impairs capsaicin-induced TRPV1 receptor tachyphylaxis, leading to sustained hyperactivity. Minocycline preincubation effectively mitigated paclitaxel-induced sensitization, restoring normal nociceptive signaling, whereas acute minocycline treatment failed to prevent these changes. ISO-1 in vitro co-incubation with paclitaxel did not affect the paclitaxel-induced changes. These findings offer novel insight into the intricate interactions among neuroinflammatory mediators, glial cell activation, and TRPV1 receptor sensitization in paclitaxel-induced neuropathic pain. The differential effects of acute versus prolonged pre-incubation minocycline application suggest the importance of sustained glial inhibition for effective outcomes and neuropathic pain management.

## Introduction

Paclitaxel (PTX), a widely used chemotherapeutic agent, is often associated with the development of chemotherapy-induced peripheral neuropathy (CIPN), characterized by neuropathic pain syndromes such as paclitaxel-acute pain syndrome and neuropathic pain [37,40,57]. Current treatments for these conditions are insufficient and need a deeper understanding of their underlying mechanisms to help create novel therapeutic methods.

Despite its peripheral distribution and much lower concentration in the central nervous system (CNS) after systemic administration, paclitaxel also affects CNS and especially dorsal root ganglion (DRG) neurons [12,16,60]. Recent findings indicate that paclitaxel activates the Toll-like receptor 4 (TLR4) pathway, similar to bacterial lipopolysaccharide (LPS), implicating TLR4 in CIPN pain modulation [9,19]. Previous studies helped to reveal the involvement of TLR4 signaling in paclitaxel-induced peripheral neuropathy, highlighting its role in spinal synaptic transmission modulation and enhanced neuronal sensitivity to TRPV1 agonists like capsaicin [1,2,26,27].

Upon activation by capsaicin or other agonists, the TRPV1 receptor functions as a non-selective cation channel, leading to calcium influx and activation of downstream kinases such as protein kinase A, protein kinase C, and calmodulin-dependent kinase II. This phosphorylation enhances TRPV1 sensitivity to agonists. To prevent over-excitation, rapid desensitization occurs via calcium/calmodulin binding to TRPV1, reducing receptor activity within seconds of continuous stimulation. With repeated or prolonged exposure, longer-term mechanisms such as PIP_2_ hydrolysis, receptor internalization, and lysosomal degradation develop, culminating in tachyphylaxis and protecting neurons from sustained excitotoxicity and potential cellular destruction [29,50].

Transient receptor potential cation channel subfamily V member 1 (TRPV1), known for its role in nociception, interacts with TLR4 and exhibits increased expression following PTX treatment [10,18,27]. This interaction potentiates TRPV1-mediated nociceptive signaling, contributing to paclitaxel-induced neuropathic pain [6]. Moreover, TRPV1 sensitization via TLR4 activation is facilitated by various kinases, including protein kinase C (PKC) and phosphatidylinositol 3-kinase (PI3K), which play pivotal roles in nociceptive transmission and neural plasticity regulation [2,13,25,53,56]. In recent years, emerging evidence has highlighted the potential therapeutic efficacy of minocycline (MX), a tetracycline antibiotic, in ameliorating neuropathic pain. Initially recognized for its antimicrobial properties, minocycline has garnered attention for its pleiotropic effects, including anti-inflammatory and neuroprotective actions. Notably, preclinical studies have demonstrated promising results suggesting that minocycline may mitigate neuropathic pain by modulating inflammatory responses, inhibiting microglial activation, and attenuating neuronal hyperexcitability [28,38].

One notable mechanism through which minocycline exerts its analgesic effects is by suppressing the activation of microglia, the resident immune cells of the central nervous system implicated in the pathogenesis of neuropathic pain. Microglial activation contributes to the release of proinflammatory cytokines, chemokines, and neurotoxic factors, perpetuating neuroinflammation and neuronal sensitization [20,59]. By inhibiting microglial activation, minocycline may disrupt this maladaptive neuroinflammatory cascade, thereby attenuating the development and maintenance of neuropathic pain.

Furthermore, minocycline has been shown to modulate various signaling pathways involved in nociceptive processing, including the mitogen-activated protein kinase (MAPK) and nuclear factor kappa B (NF-κB) pathways [49]. These pathways play pivotal roles in mediating neuroinflammation and synaptic plasticity, which are implicated in the pathophysiology of neuropathic pain. By targeting these signaling cascades, minocycline may exert multifaceted effects on neuronal function, ultimately alleviating pain hypersensitivity associated with neuropathic conditions.

Macrophage migration inhibitory factor (MIF) is widely expressed in the CNS and is involved in neuroinflammatory processes, neuronal survival, and synaptic plasticity [4,32,47]. ISO-1 is a small molecule inhibitor of MIF, a pro-inflammatory cytokine that plays a critical role in immune regulation and inflammatory responses. ISO-1 has gradually drawn significant attention for its potential therapeutic implications in various pathological conditions, particularly those involving the central nervous system (CNS) and pain pathways [34]. By inhibiting MIF activity, ISO-1 has demonstrated the ability to modulate neuroinflammation, a key factor in the pathogenesis of neurodegenerative diseases such as Alzheimer’s and Parkinson’s disease [33, 36, 41], as well as multiple sclerosis [14]. ISO-1 ability to suppress inflammatory response has also been linked to the attenuation of oxidative stress and the reduction of excitotoxicity, both of which are critical mechanisms in CNS injury and degeneration [58].

Chronic pain, particularly neuropathic pain, often involves complex interactions between the immune system and the nervous system. MIF is a prominent pain mediator due to its capacity to amplify pro-inflammatory cytokine production, enhance immune cell recruitment [23], and modulate glial cell activation within the CNS. ISO-1 should disrupt these pathways by targeting MIF, thereby mitigating central sensitization and hyperalgesia. Preclinical studies have shown that ISO-1 can reduce pain behaviors in animal models of inflammatory and neuropathic pain [15], highlighting its potential as a novel analgesic agent. Its effects are thought to be mediated through peripheral immune modulation and direct action on glial cells in the spinal cord.

In our current study, we investigated the involvement of glia activation in modulating spinal TRPV1 receptor function using an in vitro model of paclitaxel-induced neuropathic pain. We have used minocycline, a glial cell inhibitor, to block glial activation and tested its effects on the increased sensitivity of TRPV1 receptors induced by paclitaxel. We also investigated whether ISO-1 can mitigate pathological PTX effects in our in vitro model. Our findings bring new insights into the complicated, poorly understood relationship between TLR4, TRPV1 and glial activation in specific conditions of paclitaxel-induced neuropathic pain.

## Methods

### Ethical considerations

All experiments were approved by the local Institutional Animal Care and Use Committee and complied with the International Association for the Study of Pain guidelines, EU Directive 2010/63/EU governing animal experiments, and the National Institutes of Health Guide for the Care and Use of Laboratory Animals. All procedures were designed to minimize animal distress and reduce the number of animals.

### Animal models

Wistar rats (21 days old) were used for spinal cord slice preparation. The rats were housed in transparent plastic cages with soft bedding. They had unrestricted access to food and water and were maintained on a 12-hour light / 12-hour dark cycle at room temperature-controlled conditions to ensure optimal well-being.

### Chemicals and reagents

All chemicals and pharmacological agents used in the study were of analytical grade and sourced mainly from Sigma-Aldrich. Tetrodotoxin citrate (TTX) was purchased from Tocris Bioscience (Bristol, UK). Capsaicin, paclitaxel (Sigma Aldrich, USA), and ISO-1 (Tocris, UK), used for electrophysiology, were used from dimethylsulfoxide (DMSO) stock solution, with a final DMSO concentration of <0.1 % in the final solution.

### Spinal cord slices preparation

Acute spinal cord slices were prepared from naïve animals. Laminectomy, the tissue-collecting procedure, was performed following the previously described methodology [26, 43]. In brief, the animal was under deep anesthesia with 3 % isoflurane (Forane®, Abbott). Its lumbar spinal cord was removed and immersed in an oxygenated ice-cold dissection solution containing (in mM) 95 NaCl, 1.8 KCl, 7 MgSO_4_, 0.5 CaCl_2_, 1.2 KH_2_PO_4_, 26 NaHCO_3_, 25 D-glucose, 50 sucrose. The spinal cord was fixed to a vibratome stage (Leica VT1200S, Germany) in a groove between two agar blocks using cyanoacrylate glue. Acute transverse slices 300 μm thick were cut from the lumbar segment L3–L5, incubated in the dissection solution for 30 min at 35 °C, and then stored in a recording solution at room temperature (~23 °C) for at least 1 hour before further use. The recording solution (artificial cerebrospinal fluid, ACSF) contained (in mM) 127 NaCl, 1.8 KCl, 1.2 KH_2_PO_4_, 2.4 CaCl_2_, 1.3 MgSO_4_, 26 NaHCO_3_, 25 D-glucose. For the electrophysiological measurement, slices were transferred into a glass-bottomed recording chamber and perfused continuously with the recording solution at a rate of ~2.0 ml/min. During the experiment, all extracellular solutions were saturated with carbogen (95 % O_2_, 5 % CO_2_). Slices destined for incubation in minocycline were transferred 1 hour after the preparation into an incubation chamber with a recording solution containing minocycline (100 μM) before the electro-physiological recording. Minocycline was used for incubation and as an inhibitor of glial cell activation during the experiment (20 μM).

### Patch-clamp recording

Whole-cell patch-clamp recordings were made from individual superficial dorsal horn neurons visualized using a differential interference contrast (DIC) microscope Zeiss Axio Examiner A.1 (Carl Zeiss Microscopy, Germany) equipped with infrared LED illumination and an infrared-sensitive camera PL-B741EU (PixeLINK®, Canada). Patch pipettes were pulled from borosilicate glass tubing (Rückl Glass, Czech Republic) on Pipette Puller P-97 (Sutter Instruments, USA) and filled with an intracellular solution with a final resistance of 3.5–7.0 MΩ. The intracellular pipette solution contained (in mM) 125 gluconic acid lactone, 15 CsCl, 10 EGTA, 10 HEPES, 1 CaCl_2_, 2 Mg_2_ATP, 0.5 NaGTP and was adjusted to pH 7.2 with CsOH. Voltage-clamp recordings in the whole-cell configuration were performed with an Axopatch 1D (Axon Instruments, USA) amplifier and Digidata 1440A digitizer (Molecular Devices, USA) at room temperature (~23 °C). Whole-cell recordings were low-pass filtered at 2 kHz and digitally sampled at 10 kHz. The series resistance of neurons was routinely compensated by 80 % and monitored during the experiment.

AMPA-mediated miniature excitatory postsynaptic currents (mEPSC) were recorded from superficial dorsal horn neurons in laminae I and II, clamped at −70 mV in the presence of 10 μM bicuculline, 5 μM strychnine, and 0.5 μM tetrodotoxin in the bath solution. The software package pCLAMP 10.5 (Axon Instruments, USA) was used for data acquisition and later offline analysis.

**Fig. 1:**
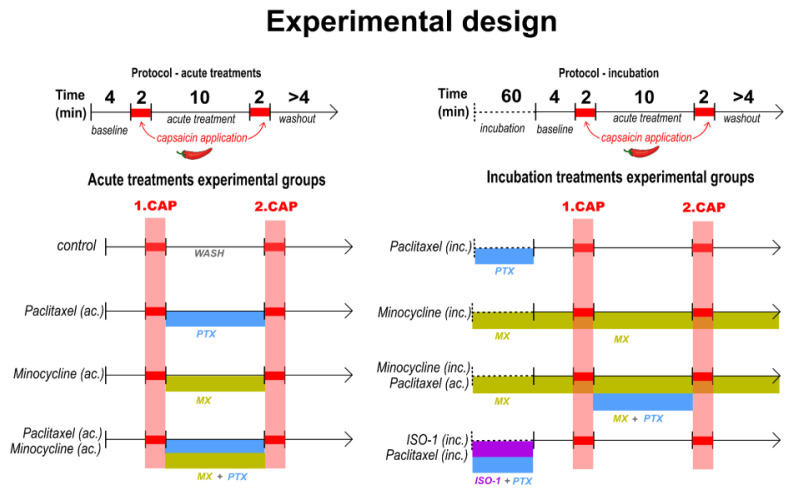
Schematic of mEPSC recording and treatment protocol

Repeated capsaicin applications on the spinal cord slice preparation were used to study the effects on presynaptic TRPV1 receptors activation and their possible tachyphylaxis. Neurons in all groups were tested with two capsaicin applications. Acute treatment or prior incubation drug-induced effects were evaluated using four experimental groups for each design (depicted in Fig. 1). The acute effect study included a control group (Ctrl), where only the recording solution was applied during the experiment; acute paclitaxel-treated (PTX ac.), where paclitaxel was applied acutely; ac. minocycline (MX ac.), where only acute minocycline was applied; and group where acute paclitaxel treatment was combined with minocycline (MX ac.+PTX ac.). The drug incubation-induced effects was assessed using paclitaxel incubation group (PTX inc.); group recorded after incubation with minocycline only (MX inc.); minocycline combined with acute paclitaxel (MX inc.+PTX ac.), where slices were first incubated in minocycline and tested with acute paclitaxel; and group of slices co-incubated in ISO-1 with paclitaxel (PTX+ISO-1 inc.).

The effect of paclitaxel (50 nM) application on tachyphylaxis of the second capsaicin response was studied as a change of mEPSCs frequency. As shown in Figure 1, recording of mEPSC began ~4 min after the whole-cell access when the recorded current had reached a steady state. After recording the control segment / basal activity (4 min), capsaicin (0.2 μM) was applied twice for 2 min with a 10 min interval between the applications. In the control groups, only the recording solution was applied between the first and the second capsaicin application. Total continuous mEPSC recording was at least 24 minutes long for each neuron. Paclitaxel (50 nM), minocycline (100 μM), or paclitaxel with minocycline were applied in acute mEPSC recordings. Paclitaxel (50 nM), minocycline (100 μM), or ISO-1 (50 μM) with paclitaxel (50 nM) were used for minimally 60 minutes of incubation of spinal cord dorsal horn slices to investigate pretreatment influence on repeated capsaicin applications. Minocycline (20 μM) was applied during recordings in both incubation experiments to maintain its complex but possibly temporary effects on microglial cells.

The mEPSCs were recorded from superficial dorsal horn neurons in spinal cord slices, focusing on the frequency and amplitude of AMPA receptor-mediated inward currents during repeated capsaicin (200 nM) applications. To assess the potential effects of paclitaxel, minocycline, and ISO-1 treatments on mEPSC, eight groups of electrophysiological experiments were organized into two main categories: acute treatments (10-minute applications of paclitaxel, minocycline, or their combination between the two capsaicin applications) and prior incubation treatments (duration between 60 – 90 minutes) with paclitaxel (50 nM), minocycline (100 μM), or ISO-1 (50 μM) in combination with paclitaxel (50 nM) as shown above. The recording protocol consisted of 4 minutes of initial (baseline) mEPSC recording, followed by 2-minute applications of capsaicin (200 nM). A 10-minute window between the capsaicin applications was used in all the different treatment conditions.

### Data and analysis

For experiment analysis, only recordings with capsaicin-evoked responses were included. Data segments of 1–2 min duration were evaluated (p-Clamp 10) for each experimental condition. The frequency and amplitude analysis included only mEPSC with an amplitude of 5 pA or greater (which corresponded to at least double the recording noise level). All summary data are expressed as a mean ± standard error of the mean (SEM). Prism software (USA) was used for statistical analysis. One-way analysis of variance (ANOVA) followed by the Dunnett post-hoc test (multiple comparison procedure versus control) or all pairwise comparison procedure (Tukey’s test) was used to analyze differences between groups with normal data distribution. We used one-way repeated measures ANOVA to test differences in one group during the treatment. We used two-way repeated measures ANOVA to compare different conditions in consequential time points of recordings. A non-parametric Wilcoxon Signed Rank Test was used to compare standardized data with non-normal distribution. The criterion for statistical significance was a p < 0.05.

## Results

### Tachyphylaxis of the capsaicin responses

**Fig. 2:**
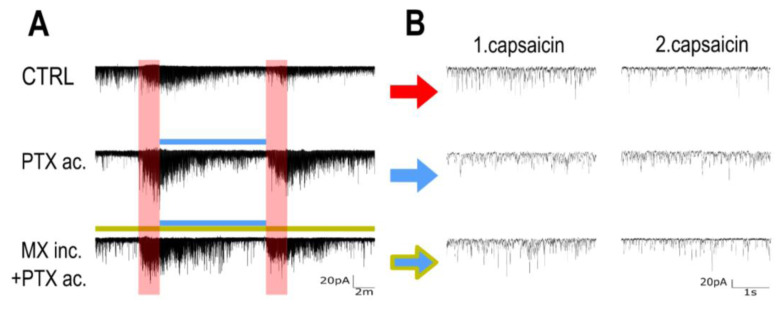
Representative Examples of mEPSC Recordings. (**A**) A representative recording of dorsal horn neurons mEPSCs from control, acute paclitaxel, and paclitaxel post-minocycline incubation groups. Colored lines represent corresponding drug applications (red bars are capsaicin, the blue line is paclitaxel, and the olive line represents a continuous application of minocycline following incubation). (**B**) 4-second segments of the corresponding mEPSC recordings selected at the end of the 2-minute first or second CAP application, when the activity was the highest.

**Fig. 3:**
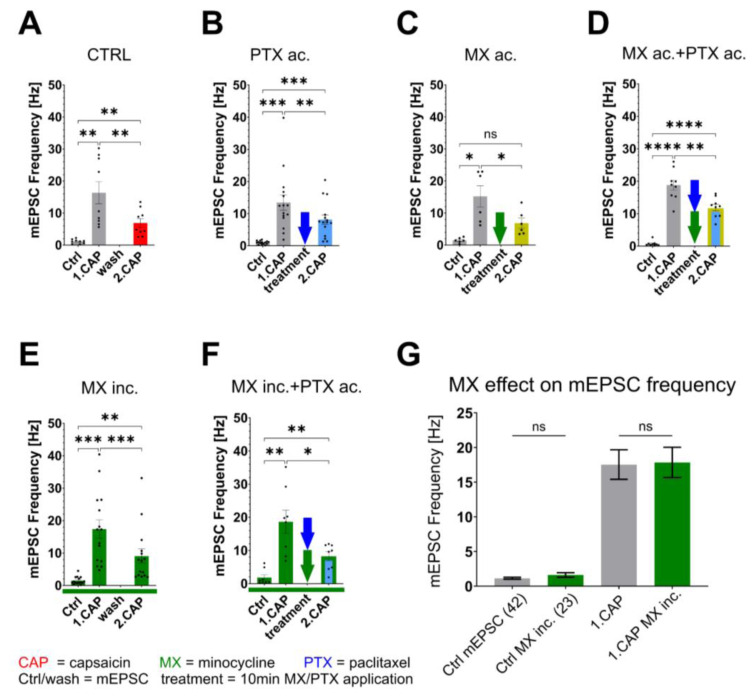
The Effects of Different Treatments on Absolute mEPSC Frequency of Repeated Capsaicin Applications. Mean mEPSC frequency (± SEM) for baseline Ctrl, first and second CAP applications are displayed together with individual values (black dots) and different treatment conditions marked with corresponding arrows. mEPSC frequency significantly increased with the first CAP application. It was significantly different from the 2nd CAP responses with all the treatments (**A**) CTRL (n=12), (**B**) acute paclitaxel (n=15), (**C**) acute minocycline alone (n=6), (**D**) acute application of paclitaxel and minocycline (n=9), (**E**) Incubation with minocycline only (n=8), (**F**) incubation with minocycline and acute application of paclitaxel (n=11). (**G**) There was no effect of the minocycline incubation when compared with the control recordings in baseline mEPSC (p=0.97), or during the first CAP application (p=0.92). (For A–F, two-way ANOVA with Tukey post-hoc test was used; F (1.49; 114.3) = 161.5; P < 0.0001); * p<0.05; **p<0.01; *** p<0.001; **** p<0.0001), for G, unpaired two-tailed T-tests with Welch’s correction were used for different SDs (ns p> 0.05).

**Fig. 4:**
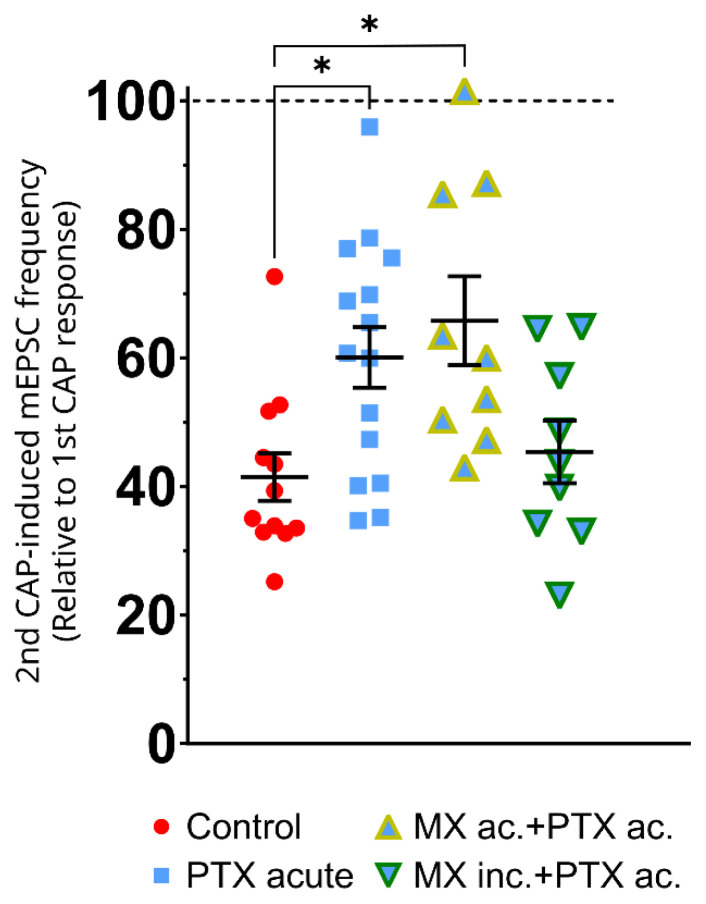
Paclitaxel and Minocycline Modulation of Tachyphylaxis within Repeated Capsaicin Application. mEPSC frequency was significantly reduced during the 2nd capsaicin application when compared to the 1st one under control conditions (41.46±3.69 % of the 1st CAP response). The mEPSC frequency during the 2nd CAP application significantly increased (60.08±4.70 %; p=0.03) after acute paclitaxel treatment and after acute co-application of paclitaxel and minocycline (65.79±6.88 %; p=0.01). However, pre-incubation with minocycline effectively mitigated paclitaxel-induced tachyphylaxis of the 2nd CAP response. Differences between groups were analyzed using one-way ANOVA with Tukey post-hoc test (F (3; 41) = 5.13; P = 0.004; *p < 0.05).

Repeated capsaicin applications were used to study TRPV1 receptor tachyphylaxis and its effect on mEPSCs in superficial dorsal horn neurons in spinal cord slices. The representative recording under the control conditions (Fig. 2, row CTRL) shows the whole progression of the mEPSC activity during the experiment. The frequency of the capsaicin-evoked events after the second CAP application was visibly smaller. The baseline mEPSC frequency (1.11±0.23 Hz) dramatically increased during the first capsaicin application (16.34±3.48 Hz), whereas the second capsaicin application response was significantly reduced to 6.89±1.37 Hz (p=0.008, Fig. 3A). As the mEPSC amplitudes remained similar (n.s.; mean amplitudes were 19.73 pA in baseline, 23.15 in 1.CAP, and 18.13 in 2.CAP), the pronounced reduction in mEPSC frequency suggested primarily presynaptic modulation of TRPV1 receptor function. The second capsaicin response was reduced to 41.46±3.69 % of the first response (Fig. 4), highlighting the expected attenuation of synaptic activity after the second CAP application under control conditions. The results from the control group show a strong tachyphylaxis of the TRPV1 receptors after repeated CAP application.

### Effects of paclitaxel on mEPSCs in spinal cord dorsal horn neurons

#### Effect of acute application of paclitaxel on capsaicin-induced tachyphylaxis

The alteration of capsaicin-induced tachyphylaxis mediated by acute paclitaxel treatment was evaluated next. The first CAP application in this case resulted in a mEPSC frequency of 13.44±1.26 Hz and decreased to 8.14±1.26 Hz during the second CAP (p=0.002, Fig. 2 and Fig. 3B). Compared to the control experiment, the relative mEPSC response to the second capsaicin application was higher (60.08±4.70 %; p=0.03, Fig. 4). These results demonstrate paclitaxel-induced TRPV1 sensitization by acute paclitaxel treatment and reduced tachyphylaxis.

#### Effects of minocycline on paclitaxel-induced sensitization

Acute application of minocycline alone had minimal impact on capsaicin-induced tachyphylaxis. The mEPSC frequency decreased from 15.18±3.30 Hz to 6.83±1.57 Hz (p=0.02, Fig. 3C), similar to control conditions. This corresponds to a relative decrease of the 2nd CAP response compared to the first CAP comparable to the control situation (41.46±3.69 %, Fig. 4). These results indicated that acute minocycline treatment did not alter the TRPV1 tachyphylaxis evoked by CAP. Incubation of spinal cord slices with minocycline alone also did not have a significant effect on the mEPSC frequency. The first capsaicin application increased the mEPSC frequency to 17.41±2.85 Hz, whereas the second capsaicin application-induced response was significantly lower (9.06±2.21 Hz, p=0.0007, Fig. 3E), closely resembling the control conditions. The relative second CAP response remained at 45.39±4.86 % of the first one, indicating that minocycline incubation alone did not significantly modify TRPV1 receptor tachyphylaxis (Fig. 4). The lack of minocycline incubation effect was also confirmed by evaluation of the all neurons recorded under the control conditions (n=42) with those incubated in minocycline (n=23) before the first capsaicin application (Fig. 3G). Statistical analysis showed no significant variation (p=0.97), indicating that minocycline incubation did not alter the baseline mEPSC activity. Also, no effects were found on the mEPSC amplitudes (mean amplitudes were in baseline 22,49 pA, in 1.CAP 24,88 pA, and in 2.CAP 21,96 pA).

Acute treatment with a combination of minocycline and paclitaxel did not mitigate paclitaxel’s effects. The baseline mEPSC frequency (0.74±0.26 Hz) increased to 18.82±1.56 Hz after the first capsaicin application and was reduced to 11.9±1.02 Hz during the second CAP (p=0.006, Fig. 3D). Highly significant differences were detected between baseline and both capsaicin applications, highlighting enhanced paclitaxel-induced hyperactivity. The relative second capsaicin-induced response was higher (65.79±6.88 %; p=0.01, Fig. 4).

Conversely, minocycline incubation combined with acute paclitaxel treatment (Fig. 3F) significantly reduced the heightened activity during the second capsaicin application, demonstrating effective suppression of paclitaxel-induced TRPV1 tachyphylaxis. The second capsaicin-induced relative response was reduced to 45.39±4.86 % of the first capsaicin response, reducing the tachyphylaxis to the control level (Fig. 4).

### Effects of paclitaxel and ISO-1 on mEPSC frequency

#### Modulation of tachyphylaxis of the capsaicin response by paclitaxel pretreatment

**Fig. 5:**
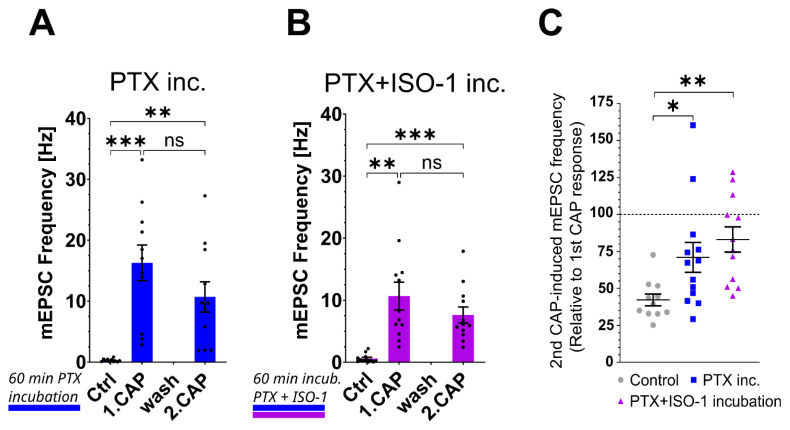
Effects of paclitaxel and ISO-1 on mEPSC Frequency after Capsaicin Application. (**A**) Incubation with paclitaxel (n=13) did not change the basal mEPSC frequency. Both capsaicin applications (CAP) induced robust mEPSC frequency increase, which was significantly different from the control but not from each other. (**B**) ISO-1, when combined with paclitaxel during incubation (n=12), did not affect the control mEPSC frequency, and CAP responses were also robust, different from the control. Mean values (±SEM) were tested with ANOVA with Tukey post-hoc test (F = 5.14; P = 0.004; **p < 0.01, ***p < 0.001). (**C**) The relative frequency of the 2nd CAP response in the percentage of the first CAP response indicated that incubation with paclitaxel increased the 2nd CAP response, and ISO-1 treatment did not affect that. Statistical significance against the control situation was assessed using ANOVA with Dunnett’s post-hoc test (F = 6.08; P = 0.0056); *p < 0.05, **p < 0.01).

Spinal cord slices were pre-incubated with paclitaxel; the first capsaicin application dramatically increased the mEPSC frequency from the baseline of 0.27±0.007 Hz up to the peak of 16.29±2.93 Hz, while the second capsaicin-induced response reached 10.7±2.48 Hz (Fig. 5A). Unlike acute paclitaxel application, no significant difference was detected between the first and second capsaicin responses (p=0.16), indicating that prolonged exposure to paclitaxel leads to persistent increase in sensitivity to CAP application. To this corresponded also analysis of the relative capsaicin responses that showed a marked reduction in CAP response tachyphylaxis. The second capsaicin response was 70.91±10.09 % of the first (p=0.037, Fig. 5C), which would be the most pronounced impairment of tachyphylaxis if compared with the previous experimental groups (Fig. 4).

#### Combined effect of ISO-1 and paclitaxel pretreatment on TRPV1 tachyphylaxis

Slices incubation with MIF inhibitor (ISO-1) and paclitaxel did not diminish the effects of paclitaxel. The first CAP response peaked at 10.68±2.25 Hz, while the second CAP application evoked an mEPSC frequency of 7.61±1.29 Hz (Fig. 5B). The relative second CAP response was 83.04±8.52 % of the first response (p=0.003 against Control, Fig. 5C), showing that ISO-1 did not affect the PTX-induced tachyphylaxis under these *in vitro* conditions.

### Time-resolved mEPSC changes during capsaicin applications

**Fig. 6:**
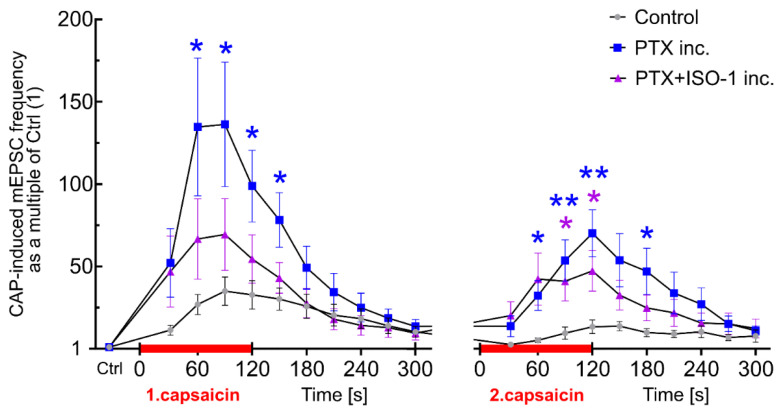
The effects of paclitaxel and ISO-1 on mEPSC frequency during repeated capsaicin applications. Frequencies of mEPSC evaluated in relation to the control frequency of each neuron (CTRL=1) give detailed information on the robustness of the responses evoked by CAP and the timeframe of these changes. Under the control conditions, CAP induced a rapid increase in mEPSC frequency, peaking at 35x baseline frequency. During the second application, the peak was almost three times lower (13x baseline), with a slow decline. In paclitaxel-treated slices, the responses to CAP were significantly higher compared to the control, reaching 136x baseline frequency during the first capsaicin application and 70x baseline during the 2nd one. Neurons recorded in slices incubated with ISO-1 and paclitaxel had lower mEPSC frequency evoked by CAP and, in this respect, did not differ from the neurons incubated with paclitaxel alone. Differences from the control group were tested for each timepoint using two-way repeated measures ANOVA against Control with Dunnett’s post-hoc tests (F (34, 680) = 11.40; P<0.0001; *p < 0.05, **p<0.01).

To further analyze the time course of capsaicin-induced synaptic responses, the mEPSC frequency data were normalized to the baseline and plotted as a function of time (Fig. 6). The relative changes provide insights into the temporal dynamics of functional TRPV1 receptor activation under different experimental conditions.

#### Tachyphylaxis of the capsaicin responses

Under control conditions, the first capsaicin application rapidly increased mEPSC frequency, peaking at 35 times baseline at 90 seconds. This was followed by a gradual decline to 10 times the baseline over the next five minutes. The second capsaicin application showed a milder peak at 14 times the baseline (150 seconds) and a decline to 8 times the baseline within five minutes, demonstrating the expected TRPV1 tachyphylaxis.

#### Paclitaxel pretreatment robustly increased capsaicin responses

In slices incubated with paclitaxel (Fig. 6), the mEPSC frequency increased much faster and higher than in control conditions. The first capsaicin application peaked at 136 times the baseline at 90 seconds, a robust increase compared to the control. This frequency elevation was significantly higher at 60 seconds and remained strongly elevated at 90 seconds, 120 seconds, and 150 seconds, despite an increasing desensitization process in the second minute of application. During the second capsaicin application, the mEPSC frequency maintained a steeper onset and prolonged elevation, peaking at 70 times its baseline at 120 seconds. This prolonged elevation suggests enhanced synaptic facilitation and impaired TRPV1 receptor tachyphylaxis, a hallmark of paclitaxel-induced neuropathic changes.

#### Effects of combined ISO-1 and paclitaxel pretreatment on TRPV1 tachyphylaxis

ISO-1 co-incubation with paclitaxel attenuated the increase in mEPSC frequency and facilitated a faster return to control levels (Fig. 6). After the first capsaicin response, ISO-1-treated slices showed a normalized decline to baseline by 180 seconds, while the second capsaicin response returned to baseline by 240 seconds. Although ISO-1 and paclitaxel-treated slices exhibited a rapid initial increase in mEPSC frequency, their second capsaicin response was visibly stronger compared to the control, and statistical differences were observed between 90 and 120 seconds of the second capsaicin response. The results in Figure 6 suggest that ISO-1 reduced the robustness of mEPSC activity evoked by PTX application in the first CAP application, as the mEPSC frequency is not significantly different from the control, but did not counteract the paclitaxel-induced reduction of CAP-evoked tachyphylaxis of the repeated application.

## Discussion

### Repeated capsaicin applications as a model of nociceptive sensitization

The repeated application of capsaicin in our study served as a model for modulation of TRPV1 receptor-mediated nociceptive synaptic transmission in the spinal cord dorsal horn. Capsaicin, an agonist of the TRPV1 receptor, is well-established in preclinical research for inducing hyperalgesia and nociceptor sensitization, which mimics pathological pain states [11,21]. Our recent findings confirm the important modulatory role of presynaptic TRPV1 receptors in the spinal cord dorsal horn for pain modulation [44–46,51] and that repeated capsaicin application induced tachyphylaxis of the evoked response, reflected in the reduction of mEPSC frequency during the second CAP application [1,2]. This aligns with previous studies showing that repeated TRPV1 activation with its agonist leads to receptor tachyphylaxis, through multiple calcium-dependent signaling pathways, dephosphorylation of the TRPV1 receptors, and their internalization [26,39,42,50].

### Paclitaxel-induced changes of tachyphylaxis in spinal cord dorsal horn neurons

Acute paclitaxel-treated slices exhibited a prolonged increase in mEPSC frequency in response to capsaicin (Fig. 4), indicating impaired desensitization mechanisms compared to the control group. This increased postsynaptic activity during both the first and second capsaicin applications highlights paclitaxel’s role in amplifying synaptic responses, potentially contributing to central sensitization and chronic pain often observed in CIPN [26,55]. Nevertheless, our findings further demonstrate that paclitaxel significantly reduces TRPV1 tachyphylaxis by enhancing mEPSC responses to repeated capsaicin applications, consistent with its established role in sensitizing synaptic activity through neuroinflammatory pathways. The significant differences between the paclitaxel-treated group and controls are consistent with prior studies demonstrating paclitaxel’s ability to increase presynaptic neurotransmitter release in nociceptive neurons via mechanisms involving TRPV1 sensitization and glial activation [2,62].

Our findings revealed that prolonged paclitaxel incubation led to a sustained increase in mEPSC frequency, supporting the idea that paclitaxel induces persistent changes in nociceptive signaling. Extended paclitaxel exposure resulted in enhancement of nociceptive signaling, supported by the robust reduction of tachyphylaxis evoked by the repeated CAP application. While under control conditions, the 2nd CAP response was only 41 % of the first one, after PAX incubation, it was 71 %. This suggests that chronic paclitaxel exposure modifies the intrinsic properties of sensory neurons and robustly changes the properties of TRPV1 receptor-mediated signaling at the spinal cord dorsal horn synapse [24].

### Minocycline as a modulator of paclitaxel-induced changes

Minocycline, a glial cell inhibitor, was evaluated for its ability to mitigate paclitaxel-induced sensitivity in the spinal cord dorsal horn in our model of paclitaxel-induced neuropathic pain. Our findings indicate that acute minocycline treatment alone had minimal effects on mEPSC frequency and TRPV1 receptor function, suggesting that its acute administration does not significantly alter nociceptive synaptic transmission. The timing and duration of minocycline administration play a crucial role in its effectiveness as an anti-nociceptive agent. In our experiments, when minocycline was applied acutely with paclitaxel, it failed to suppress paclitaxel-induced sensitization and tachyphylaxis. The tendency to increase the CAP responses could be potentially due to incomplete suppression of neuroinflammatory pathways [35].

Previous studies suggested that acute minocycline applications can transiently alter nociceptive signaling without entirely suppressing glial activation [35,61]. The failure of acute minocycline treatment to prevent paclitaxel-induced sensitization, even during in vitro conditions, suggests that short-term inhibition of glial activity is insufficient to counteract paclitaxel-induced neuropathic pain-related changes [8,26].

In contrast, pre-incubation with minocycline effectively diminished paclitaxel-induced hypersensitivity, restoring mEPSC frequency to control levels during the second capsaicin application. These findings indicate that prolonged minocycline administration attenuates neuroinflammation induced by PTX and prevents TRPV1 receptor sensitization and the associated pronociceptive activity. This effect is likely mediated through inhibition of glial activation and neuroimmune signaling leading to reduced local production of proinflammatory agents. The extended minocycline treatment thus appears to be more effective than acute treatment in counteracting paclitaxel-induced modulation of TRPV1 receptors function [22].

Our results suggest the critical role of glial activation in paclitaxel-induced synaptic plasticity and the importance of chronic inhibition of their function for effective anti-nociception. While some studies report conflicting results regarding minocycline’s efficacy in different pain models, the current findings support its use in chemotherapy-induced neuropathy when administered over a prolonged period [38, 61].

### ISO-1 as a modulator of paclitaxel-induced sensitivity

We further investigated the impact of ISO-1, a macrophage migration inhibitory factor (MIF) antagonist incubation on modulation synaptic activity using paclitaxel and the same repeated capsaicin application protocol.

Co-incubation with ISO-1 did not significantly change the responses to CAP application measured as mEPSC frequency and also did not alter the repeated CAP application-induced tachyphylaxis of the response. These results suggest that ISO-1 incubation in vitro had only a minimal modulatory effects on the TRPV1 receptors function after paclitaxel application. Interestingly, co-incubation with ISO-1 did not significantly alter the tachyphylaxis induced by repeated CAP application but reduced the overall activation by CAP after PTX incubation when analyzed as the percent change of the control activity (Fig. 6). ISO-1 is recognized for its anti-inflammatory properties, particularly in modulating glial responses [3, 5]. Previous studies have also linked MIF to neuroimmune interactions that contribute to chemotherapy-induced neuropathic pain, making ISO-1 a potential candidate for modulating glial-driven nociceptive pathways [31,52]. However, our findings suggest that ISO-1 alone may not be sufficient to fully counteract paclitaxel-induced synaptic plasticity changes. ISO-1’s neuroprotective effects may be also limited to situations with heightened sensitization conditions [17] and most likely due to activation of other mechanisms in vivo, that are not present in our in vitro model [7].

Previous studies have demonstrated that ISO-1 suppresses neuronal apoptosis, axonal injury, and glial responses, further supporting its neuroprotective and anti-inflammatory properties [52, 63]. Additionally, research has shown that ISO-1 administration significantly alleviates inflammatory responses, reinforcing its potential role in modulating immune-mediated pathologies [3]. These findings emphasize the complex interplay between neuroimmune modulation and synaptic plasticity in the context of chemotherapy-induced neuropathic pain. Future research should explore combination therapies targeting multiple pathways to achieve more effective neuroprotection and pain management [48].

### Minocycline and ISO-1 as potential modulators of CIPN

The comparative analysis of relative changes in mEPSC frequency across different treatments demonstrated that paclitaxel significantly increased nociceptive signaling to capsaicin. Minocycline-alone treatments had no significant effects on the baseline activity. However, incubation with minocycline reduced paclitaxel-induced sensitization and restored tachyphylaxis of TRPV1 receptors to repeated CAP application.

These findings highlight the critical role of glial activation in paclitaxel-induced synaptic plasticity and suggest that targeting glial-mediated modulation of nociception as a promising strategy for managing chemotherapy-induced pain. Furthermore, the partial effects of ISO-1 in vitro suggest that preventing PTX induced pathology may require modulating complex neuroimmune interactions. Minocycline, known for its glial inhibitory properties, has been shown to prevent paclitaxel-evoked allodynia also by inhibiting intraepidermal nerve fiber loss and macrophage infiltration in rats [30]. Our previous studies have indicated that paclitaxel increases nociceptive synaptic transmission via TLR4 activation in the spinal cord [26]. Therefore, targeting different neuroimmune pathways might offer enhanced treatment effects in neuropathic pain conditions [1,54]. Further studies are needed to explore the possible additive effects of combination treatments with minocycline and ISO-1 under in vivo and in vitro conditions in models of CIPN.

## Conclusions

This study confirmed pro-nociceptive pathological effects of PTX on TRPV1 receptors mediated modulation of synaptic transmission in spinal cord dorsal horn. Glial cells inhibitor minocycline and MIF inhibitor ISO-1 showed some promising potential for CIPN treatment in our in vitro model. Our findings emphasize the role of complex interaction between glial cells, neuroimmune factors, and TRPV1 receptor function in chemotherapy-induced pain and highlight potential therapeutic strategies targeting both neuroinflammatory and immune pathways. The timing, duration, and combination of neuroprotective, anti-nociceptive treatments may be critical for managing paclitaxel-induced neuropathic changes. Further research should explore the combined use of anti-inflammatory agents with specific receptor modulators to develop more effective strategies for alleviating chemotherapy-induced neuropathic pain.

## Abbreviations

ANOVA: analysis of variance
CNS: central nervous system
CIPN: chemotherapy-induced peripheral neuropathy
mEPSC: Miniature excitatory postsynaptic currents
PTX: paclitaxel
PKC: protein kinase C
MIF: Macrophage migration inhibitory factor
MX: minocycline
PI3K: phosphatidylinositol 3-kinase
DRG: Dorsal root ganglia
TLR4: Toll-like receptor 4
TRPV1: Transient receptor potential cation channel subfamily V member 1
